# Ultrasound-Guided Collagen Peptides Injections Combined with Focused Extracorporeal Shockwave Therapy and Therapeutic Exercise in Chronic Partial Common Extensor Tendon Tears at Elbow Lateral Epicondyle: A Retrospective Case Series

**DOI:** 10.3390/life16091465

**Published:** 2026-09-02

**Authors:** Nicolò Vitale, Luca Latini, Marco Di Gesù, Ilias Vurliotis, Marianna Capecci, Valerio Sebastiano Amico, Florentin Ananu Vreju, Emilio Filippucci

**Affiliations:** 1 Centro Medicina Riabilitativa “CMR”, 95031 Adrano, Italy; nicovitale91@gmail.com; 2 Centro Medico e Fisioterapia “Salute e Benessere”, 60019 Senigallia, Italy; 3 MyaRehab, 90146 Palermo, Italy; digesumarco@gmail.com; 4 Department of Experimental and Clinical Medicine, Università Politecnica delle Marche, Via Tronto 10, 60126 Ancona, Italy; ivurliotis@gmail.com (I.V.); m.capecci@staff.univpm.it (M.C.); 5 G2 Medica, 95129 Catania, Italy; valeriosebastianoamico@gmail.com; 6 Department of Rheumatology, University of Medicine and Pharmacy of Craiova, 200349 Craiova, Romania; florentin.vreju@umfcv.ro; 7 Rheumatology Unit, Department of Clinical and Molecular Sciences, Polytechnic University of Marche, 60035 Jesi, Italy

**Keywords:** lateral epicondylitis, tennis elbow, common extensor tendon, lateral elbow tendinopathy, partial tendon tear, collagen peptides, hydrolyzed collagen, ultrasound-guided injection, extracorporeal shockwave therapy, therapeutic exercise

## Abstract

Background: Lateral elbow tendinopathy (LET) is a common upper-limb musculoskeletal disorder causing pain during gripping activities, reduced work capacity, and functional limitation. LET is currently considered a predominantly degenerative condition characterized by collagen disorganization, extracellular matrix changes, neovascularization, and impaired tendon healing. Objective: To evaluate short-term clinical and functional outcomes of a multimodal protocol including focused extracorporeal shock wave therapy (f-ESWT), ultrasound-guided intralesional injection of low-molecular-weight peptides (LWPs) derived from hydrolyzed bovine collagen and therapeutic exercise in patients with chronic LET unresponsive to standardized conservative therapy. Methods: This retrospective observational study included 11 screened patients; 10 completed follow-up. Eligibility required age ≥ 18 years, symptoms ≥ 3 months, positive provocation tests, ultrasound-confirmed common extensor tendon pathology, and incomplete response to prior treatment. All patients received f-ESWT before ultrasound-guided LWPs injection and performed progressive loading exercises. Outcomes included Patient-Rated Tennis Elbow Evaluation (PRTEE), grip strength, and clinical provocation tests at baseline (T0; Day 0) and at T1 (Day 51, 30 days after injection). Results: PRTEE pain improved by 31.2 points (95% CI 22.2–40.2; Cohen’s d_z = 2.48) and PRTEE function by 32.6 points (95% CI 26.7–38.5; d_z = 3.95). Mean maximal strength and maximal strength improved by 4.61 kg (95% CI 2.89–6.32) and 4.02 kg (95% CI 2.36–5.68), respectively. Clinical test severity decreased by an estimated 2.5 points (95% CI 2.0–3.0). No adverse events were reported. Conclusions: Patients showed clinically meaningful short-term improvements in pain, function, and strength following the sequential multimodal management strategy. However, the retrospective design, small sample, lack of control group, and short follow-up preclude causal attribution and require confirmation in prospective controlled studies.

## 1. Introduction

Lateral elbow tendinopathy (LET) is one of the most common overload disorders affecting the upper limb in outpatient musculoskeletal practice [1]. The prevalence of elbow injuries appears to be highest among individuals aged 50 to 59 years, with a slight predominance observed in the male population (55.2%) [2]. Clinically, patients usually report pain over the elbow lateral epicondyle, reduced confidence during gripping tasks, difficulty performing resisted wrist extension, and a progressive limitation in work, sport, or domestic activities. Histopathological studies have shown that the condition is not primarily inflammatory, but rather degenerative, with collagen disorganization, angiofibroblastic hyperplasia, increased ground substance, and variable degrees of neovascularization [2,3]. This biological background helps explain why symptoms may become persistent and why some patients do not respond adequately to conventional symptomatic treatment [4].

From a practical physiatric perspective, LET is not only a source of pain but also a disorder of tendon load tolerance. For this reason, management should not be limited to transient pain control, but should ideally aim at restoring the tissue’s capacity to sustain progressively increasing mechanical demands. Exercise therapy remains the cornerstone of conservative management, particularly when structured around progressive loading principles [5,6,7,8,9]. F-ESWT is also widely used and may contribute through mechanotransduction, modulation of pain pathways, stimulation of neovascular response, and support of tissue remodeling [10,11,12]. However, even when these approaches are correctly prescribed, a subgroup of patients continues to show incomplete recovery.

In recent years, biological and regenerative approaches have attracted increasing interest in tendinopathy care [13,14,15,16]. Among them, collagen-based injective therapies appear particularly appealing in chronic degenerative conditions, because they may provide both biochemical stimuli and structural support for tendon healing. Hydrolized collagen peptides may favor fibroblast activity, extracellular matrix synthesis, and tendon remodeling, thus acting on mechanisms that are directly relevant to chronic tendon degeneration [15,16,17,18]. However, despite this biological rationale, the clinical evidence supporting injectable collagen-derived peptides in LET remains limited. Available data are primarily derived from small uncontrolled observational or pilot studies, and high-quality comparative evidence is currently lacking.

In our clinical experience, these therapies are especially interesting when integrated within a broader rehabilitation strategy rather than used as stand-alone solutions.

Ultrasound guidance is key element for performing injection treatment according to best clinical practice. Beyond improving needle placement accuracy, ultrasound allows a real-time definition of tendon morphology, localization of the structural lesion, and a targeted intralesional approach [19,20]. This diagnostic–therapeutic integration is especially valuable in partial common extensor tendon (CET) tears, where symptom distribution and structural damage do not always fully overlap on clinical examination alone.

The rationale behind the present study was therefore to characterize, in a real-world retrospective cohort, the short-term clinical and functional changes observed following a pathophysiology-driven sequential multimodal strategy combining f-ESWT, ultrasound-guided intralesional LWPs injection, and therapeutic exercise in patients with chronic LET and partial CET tears.

## 2. Materials and Methods

The study was conducted in accordance with the principles of good clinical practice. Due to the retrospective and observational nature of the study, based on anonymized clinical data collected during routine practice, formal ethics committee approval was not required according to local procedural standards. Written informed consent for treatment and use of anonymized data for scientific purposes was obtained from all patients.

This retrospective case series included patients with chronic LET and partial CET tears. A total of 11 patients met the inclusion criteria and entered the study; 10 provided complete T0 and T1 data and were included in the final analysis.

The study population was defined according to clinical and imaging-based eligibility criteria summarized in Table 1.

**Figure 1:**
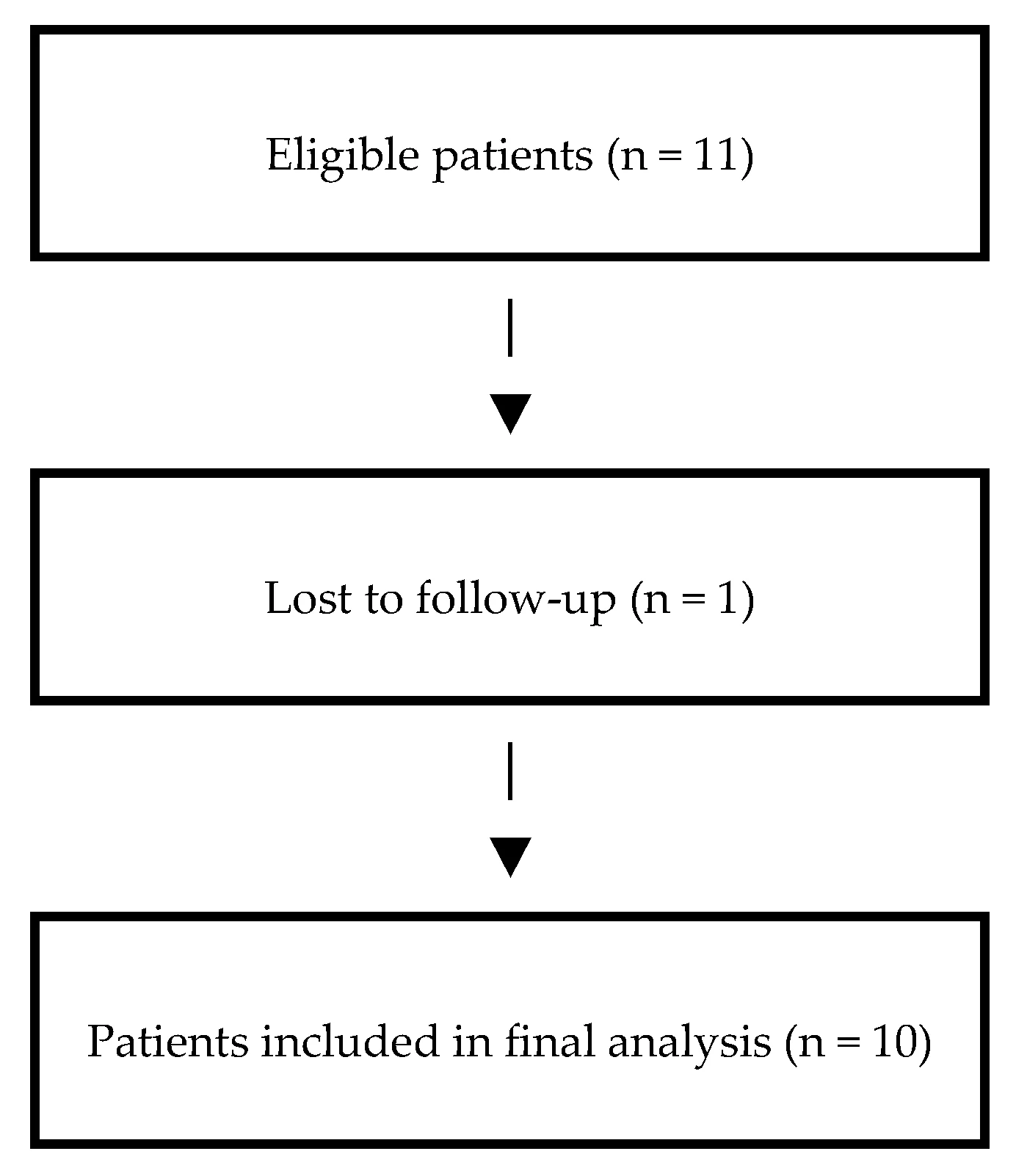
Patients flow diagram.

The patient selection process, including screening, eligibility assessment, loss to follow-up, and final inclusion in the analysis is summarized in Figure 1.

F-ESWT (Storz, Duolith^®^ SD, Tägerwilen, Switzerland) was administered in two sessions at weekly intervals, with 2000 pulses, 4 Hz, delivered at an energy flux density of 0.20 mJ/mm^2^, using ultrasound-assisted targeting of the pathological tendon area. Therapeutic exercise was based on progressive loading of the wrist extensor musculotendinous unit, with the aim of progressively restoring tendon load tolerance and upper-limb function. Exercise selection and loading were individually adapted according to symptom irritability and the patient’s clinical response to loading, progressing from lower-irritability tasks toward progressively more demanding resisted wrist-extension activities. The rehabilitation program was performed under the supervision of an experienced physiotherapist, who provided education regarding appropriate tendon loading, activity modification, and avoidance of excessive symptom-provoking activities. This loading strategy was consistent with contemporary recommendations for lateral elbow tendinopathy, which support resisted isometric, concentric, and/or eccentric wrist-extensor exercise and a phased reintroduction of mechanical stress [21,22,23]. Because of the retrospective nature of the study, detailed standardized exercise dosage parameters were not uniformly available in the clinical records; therefore, the number and intensity of the exercises performed may have varied from patient to patient, based on the guiding principles previously described.

Two weeks after last f-ESWT treatment, patients received an ultrasound-guided intralesional injection composed of 1 mL of LWPs derived from hydrolyzed bovine collagen (Tiss’You, Domagnano, San Marino Republic, 5 mg/mL). The procedure was performed under sterile conditions using an Esaote MyLAB™X75 (Esaote S.p.A. Genoa, Italy) and multifrequency linear 4–15 MHz probe and in-plane needle visualization, with careful targeting of the structural tendon lesion. In our clinical workflow, the aim of the injection was not simply analgesic, but rather to provide local biological support to a tendon that had already entered a mechanically guided rehabilitation pathway.

**Figure 2:**
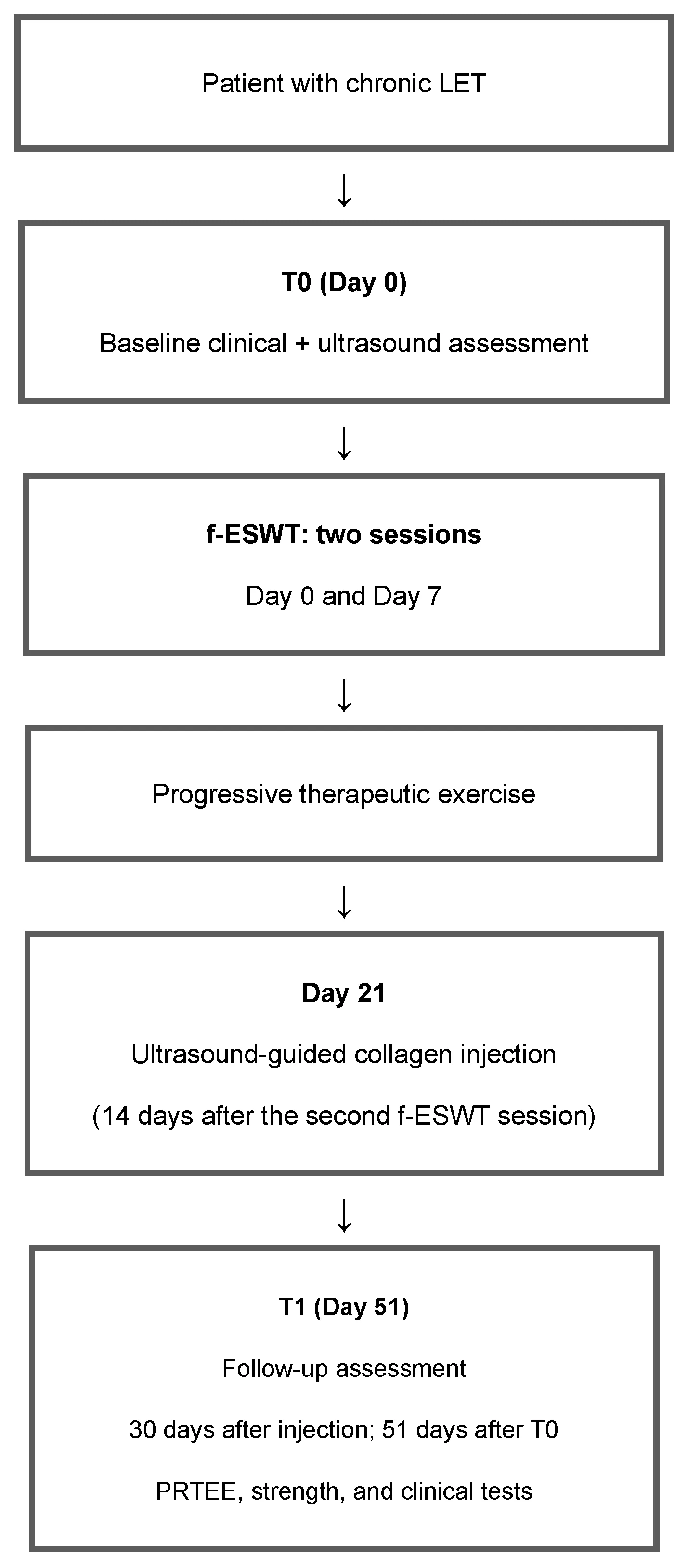
Treatment protocol flowchart and study timeline. T0 was performed on Day 0 immediately before the first f-ESWT session; T1 was performed on Day 51, corresponding to 30 days after the injection and 51 days after T0. Abbreviations: LET, lateral elbow tendinopathy; f-ESWT, focused extracorporeal shock wave therapy; PRTEE, Patient-Rated Tennis Elbow Evaluation.

The stepwise treatment algorithm used in this study, including baseline clinical and ultrasound assessment, f-ESWT, progressive therapeutic exercise, ultrasound-guided collagen injection, and Day-51 follow-up evaluation (30 days post injection), is summarized in Figure 2. The Day-51 follow-up, performed 30 days after injection, was selected to assess early clinical responsiveness to the multimodal protocol. In our clinical experience, this interval is generally sufficient to detect clinically meaningful changes in pain, function, and strength and to identify patients with persistent symptoms or apparent early treatment refractoriness. This time point was not intended to assess long-term tendon healing or structural remodeling.

Baseline assessment (T0) was performed immediately before the first f-ESWT session on Day 0. The second f-ESWT session was delivered on Day 7, followed by the ultrasound-guided collagen injection on Day 21 (14 days after the second f-ESWT session). Follow-up assessment (T1) was performed on Day 51, corresponding to 30 days after the injection and an exact interval of 51 days between T0 and T1.

Clinical evaluation was performed at baseline (T0; Day 0, immediately before the first f-ESWT session) and at follow-up (T1; Day 51, 30 days after injection). The primary outcomes were changes in patient-reported pain and function between T0 and T1, assessed using the PRTEE pain and function subscales [8,9]. For the exploratory individual responder analysis, the total PRTEE score was calculated as the sum of the pain and function subscales (range 0–100). A recently reported minimal clinically important difference (MCID) for the total PRTEE score in patients with LET is 14.8 points at 3-month follow-up [10]. Secondary outcomes included changes in objective wrist extensor strength assessed by dynamometry and changes in severity of the Cozen, Mill and Maudsley provocation tests. To better capture change in signs irritability over time, these tests were graded using a four-level semi-quantitative scale (Table 2).

The four-level semi-quantitative ordinal scale was developed by the authors specifically for the present retrospective study and was not adapted from a previously published or validated instrument. All clinical and dynamometric assessments were performed by the same experienced physiatrist at both baseline (T0) and follow-up (T1).

This approach was chosen because, in everyday clinical practice, a binary positive/negative classification may be less sensitive to change.

Dynamometric assessment was performed using a Mustec Handheld Dynamometer Force Evaluator V3 (Almere, The Netherlands) to quantify resisted wrist extension strength. Maximum strength and mean maximal strength values were recorded in kilograms and interpreted in relation to side-to-side difference and clinical recovery [24,25]. In our practice, the combination of patient-reported outcome measures, provocative clinical testing, and objective strength testing was considered more informative than relying on any single outcome domain alone.

Statistical analyses were performed on the 10 patients with complete paired baseline (T0) and follow-up (T1) data; no missing-data imputation was performed. Continuous variables were expressed as mean ± standard deviation (SD). Within-subject differences were defined so that positive values represented clinical improvement (T0 − T1 for PRTEE scores and T1 − T0 for strength and duration measures). Their distributions were examined at the individual-patient level using patient-level difference plots and assessed descriptively using the Shapiro–Wilk test. Given the limited power of normality tests with only 10 paired observations, a non-significant Shapiro–Wilk result was not interpreted as evidence that normality had been established. For continuous outcomes, T0 and T1 were compared using two-sided paired Student’s *t*-tests. Effect estimates are reported as mean paired changes with corresponding 95% confidence intervals (95% CIs) calculated from the t distribution. Standardized within-subject effect sizes were calculated using Cohen’s d_z, defined as the mean paired change divided by the SD of the individual paired change scores, consistent with the standard definition for paired/within-subject comparisons [26]; 95% CIs for d_z were derived from the noncentral t distribution. Exact two-sided Wilcoxon signed-rank tests were additionally performed as sensitivity analyses for all continuous outcomes, with median paired changes, interquartile ranges (IQRs), observed ranges, and signed-rank statistics reported. For the exploratory individual-level PRTEE responder analysis, clinically meaningful improvement was defined as a reduction of at least 14.8 points in the total PRTEE score [10]. The responder proportion was reported with an exact Clopper–Pearson 95% binomial confidence interval. Because the clinical provocation-test severity score was ordinal, it was analyzed using the two-sided Wilcoxon signed-rank test; the magnitude of change is reported as the Hodges–Lehmann estimate with its 95% CI. Associations among clinical and functional variables at T1 were explored using Spearman’s rank correlation coefficient (rho), with 95% CIs calculated using the Bonett–Wright Fisher-transformation method. Given the very small sample size (n = 10), correlation analyses were considered exploratory and hypothesis-generating, and no causal or predictive inference was made. Because these correlation analyses were exploratory, no adjustment for multiple comparisons was applied and their *p*-values were interpreted descriptively. All statistical tests were two-sided, with statistical significance set at *p* < 0.05. Analyses were performed using Python 3.13 and SciPy 1.17.0.

## 3. Results

A total of 11 patients were investigated. One patient did not attend the follow-up visit and was excluded from the final analysis, resulting in a sample of 10 patients. Baseline characteristics of the patient who did not attend the follow-up visit were as follows: male sex, age 42 years, body weight 75 kg, height 175 cm, and baseline total PRTEE score 80/100 (pain subscale 41/50 and function subscale 39/50). The affected side is the dominant one (Table 3). Overall, the treatment protocol was well tolerated and no adverse events were reported.

A marked reduction in PRTEE scores was observed after treatment. PRTEE pain score decreased from 44.3 ± 2.5 at baseline to 13.1 ± 11.2 at follow-up, corresponding to a mean paired improvement of 31.2 points (95% CI 22.2–40.2; Cohen’s d_z = 2.48, 95% CI 1.18–3.76; *p* < 0.0001). PRTEE function score decreased from 41.5 ± 3.8 to 8.9 ± 7.6, corresponding to a mean paired improvement of 32.6 points (95% CI 26.7–38.5; d_z = 3.95, 95% CI 2.05–5.84; *p* < 0.0001).

**Figure 3:**
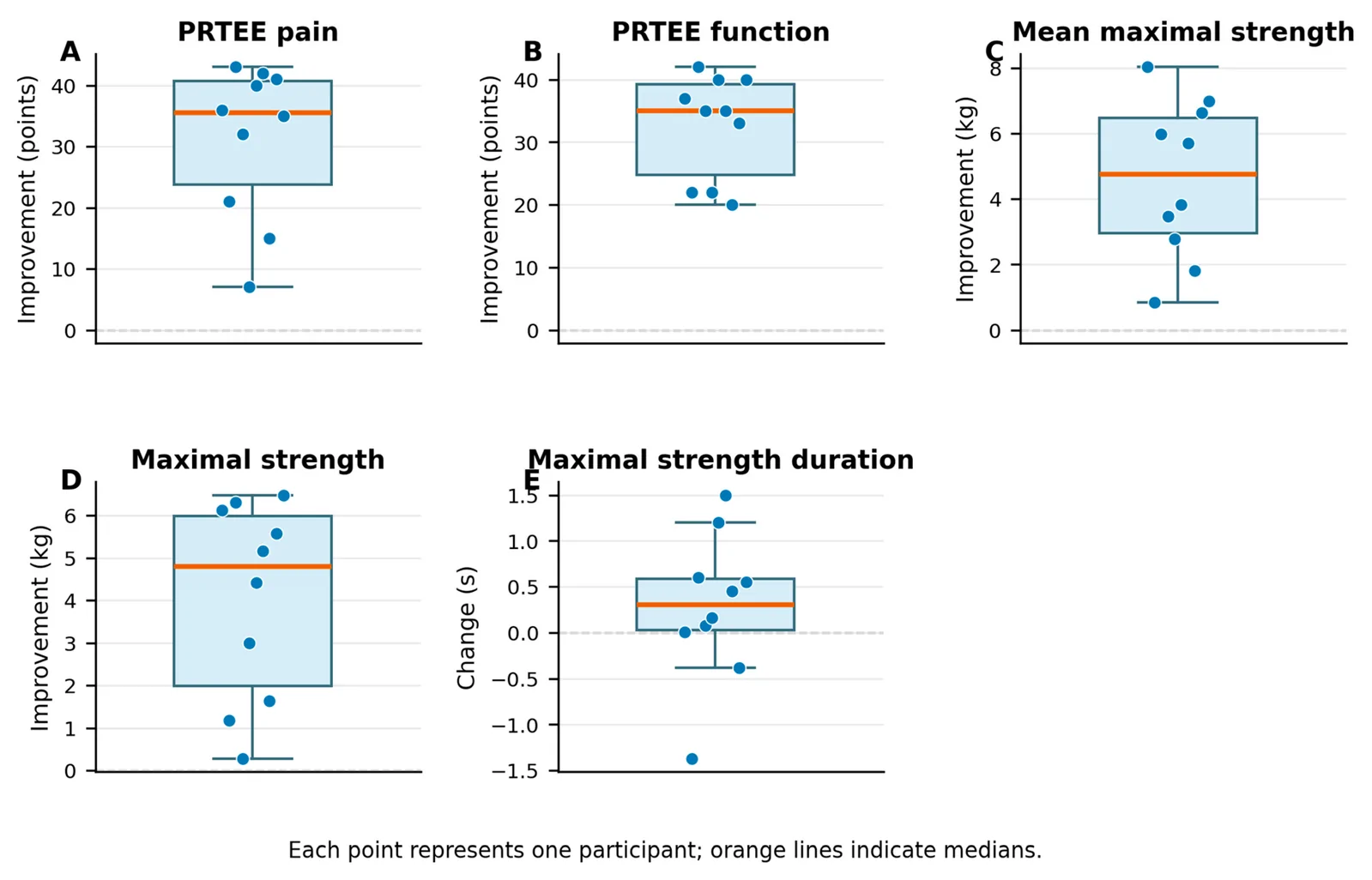
Individual within-subject paired differences for the continuous outcomes. Differences were defined so that positive values indicate clinical improvement. Each point represents one participant; the orange horizontal line indicates the median, and the dashed horizontal line indicates no change.

Inspection of the individual paired differences (Figure 3) showed improvement in all 10 patients for PRTEE pain, PRTEE function, mean maximal strength, and maximal strength, whereas changes in maximal strength duration were heterogeneous. Shapiro–Wilk W (*p*) values for the paired differences were 0.855 (0.067), 0.856 (0.068), 0.955 (0.732), 0.887 (0.158), and 0.958 (0.763), respectively. Because of the limited power of normality testing with n = 10, these values were interpreted descriptively. Exact two-sided Wilcoxon sensitivity analyses confirmed improvements in PRTEE pain (median 35.5 points, IQR 23.75–40.75; W = 0; *p* = 0.002), PRTEE function (35.0 points, IQR 24.75–39.25; W = 0; *p* = 0.002), mean maximal strength (4.77 kg, IQR 2.96–6.47; W = 0; *p* = 0.002), and maximal strength (4.79 kg, IQR 1.98–5.99; W = 0; *p* = 0.002). Maximal strength duration remained non-significant (median change 0.31 s, IQR 0.03–0.59; W = 13; *p* = 0.160). These sensitivity results were concordant with the paired *t*-tests.

**Figure 4:**
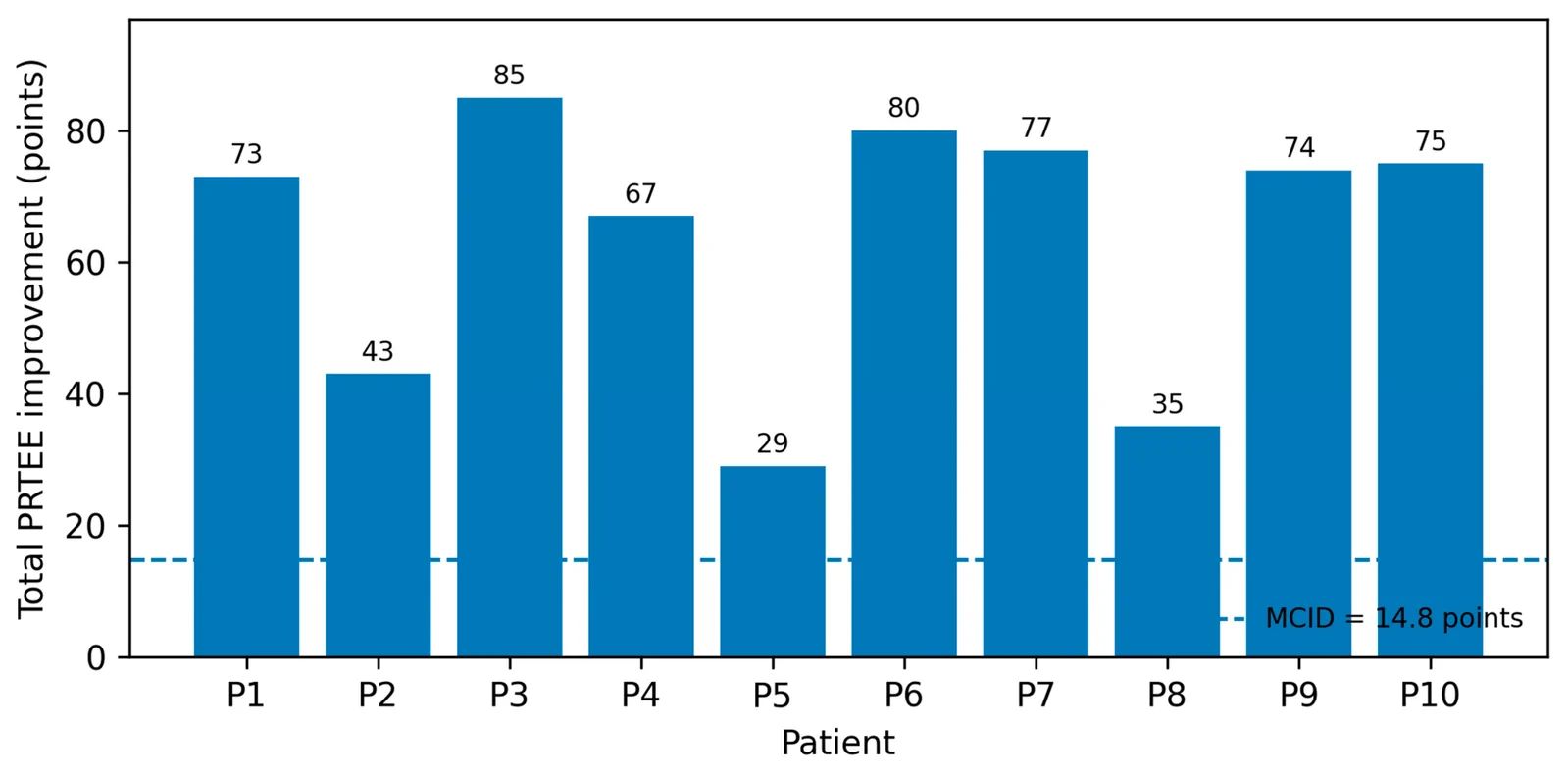
Individual improvement in total PRTEE score from baseline (T0; Day 0) to follow-up (T1; Day 51, 30 days after injection). Positive values indicate improvement. The dashed horizontal line represents the 14.8-point MCID reported by Young et al. [10]. All 10 patients exceeded this threshold.

At the individual-patient level, total PRTEE scores improved in all 10 patients. Using the 14.8-point MCID threshold reported by Young et al. [10], all 10 patients met the responder criterion (100%; exact 95% CI 69.2–100.0%). Individual reductions in total PRTEE ranged from 29 to 85 points. Individual patient values and responder status are reported in Table 4, and the distribution of individual changes relative to the MCID threshold is illustrated in Figure 4.

Dynamometric assessment also showed substantial within-subject changes. Mean maximal strength changed from −4.80 ± 1.60 kg at T0 to −0.19 ± 1.48 kg at T1, corresponding to a mean improvement of 4.61 kg (95% CI 2.89–6.32; d_z = 1.92, 95% CI 0.84–2.98; *p* = 0.0002). Maximal strength changed from −4.67 ± 2.01 kg to −0.66 ± 1.30 kg, corresponding to a mean improvement of 4.02 kg (95% CI 2.36–5.68; d_z = 1.73, 95% CI 0.71–2.72; *p* = 0.0004). No clear change was observed in maximal strength duration (mean change 0.28 s, 95% CI −0.30 to 0.86; d_z = 0.35, 95% CI −0.30 to 0.98; *p* = 0.300).

Clinical examination findings also improved. The clinical provocation-test severity score decreased from a median of 3.0 (IQR 3.0–3.0) at baseline to 0.5 (IQR 0.0–1.0) at follow-up. The Hodges–Lehmann estimated reduction was 2.5 points (95% CI 2.0–3.0; Wilcoxon W = 0; *p* = 0.002), with improvement observed in all analyzed patients.

Exploratory correlation analysis at follow-up showed a positive association between PRTEE pain and PRTEE function (Spearman rho = 0.881, 95% CI 0.467–0.978; *p* = 0.0008). An inverse association was observed between PRTEE pain and mean maximal strength (rho = −0.659, 95% CI −0.923 to 0.027; *p* = 0.038), while the association between PRTEE function and mean maximal strength was rho = −0.606 (95% CI −0.907 to 0.104; *p* = 0.064). Given the very small sample size and the resulting wide confidence intervals, these correlation estimates should be considered exploratory and hypothesis-generating; no causal or predictive inference was drawn from them.

The tabulated statistical summary, including effect estimates, distributional checks, exact Wilcoxon sensitivity analyses, and 95% confidence intervals, is reported in Table 4 and Table 5.

## 4. Discussion

This study describes a pathophysiology-driven sequential multimodal strategy integrating mechanical and biological interventions for chronic LET with structural tendon damage detected by US. Within this framework, patients showed clinically meaningful short-term improvements in pain, function, and strength following the multimodal management strategy. Importantly, these changes were observed in a population characterized not only by persistent symptoms but also by imaging-confirmed tendon degeneration, a subgroup in which standard conservative approaches often show limited effectiveness [2,3,4].

The rationale underlying this strategy is not merely the combination of different therapeutic modalities, but their structured sequencing according to tendon pathophysiology. F-ESWT was initially used to provide a mechanical and biological stimulus, potentially enhancing local tissue responsiveness through mechanotransductive and angiogenic mechanisms [10,11,12]. This phase was followed by progressive therapeutic exercise aimed at restoring tendon load tolerance and functional capacity, considering that no injective treatment should be assumed sufficient by itself in chronic LET if the tendon is not progressively re-exposed to functional loading [5,6,7,8,9]. Beyond its mechanical effects on tendon adaptation, therapeutic exercise may also contribute to improving sensorimotor integration and adaptive motor control. Contemporary models increasingly describe pain and motor behavior as dynamic processes shaped by prediction, behavioral adaptation, and continuous interaction between sensory input and motor output rather than purely peripheral nociceptive mechanisms alone [27]. Within this perspective, progressive exercise performed after f-ESWT may facilitate a more favorable neuromuscular environment before the administration of the biological injective treatment. This concept is consistent with contemporary rehabilitation paradigms in which recovery is viewed not simply as symptom reduction, but as progressive restoration of movement confidence, motor adaptability, and functional performance through active exposure to meaningful mechanical tasks [5,6,7,8,9]. In this model, therapeutic exercise was not conceived as a final adjunctive phase, but rather as an intermediate functional reconditioning step potentially capable of improving tissue readiness before biological support. Finally, US-guided intralesional injection of LWPs derived from hydrolyzed collagen was introduced as a targeted biological support intended to promote extracellular matrix remodeling in a tendon that had already undergone mechanical and functional reconditioning [15,16,17,18].

This sequencing may be particularly relevant in chronic partial CET tears, where structural disorganization, impaired healing capacity, and altered load tolerance coexist. LET is currently understood as a degenerative tendon disorder characterized by collagen disorganization, angiofibroblastic changes, and failed healing response rather than a classical inflammatory process [2,3,10]. In this context, isolated symptomatic treatments may be insufficient, whereas a strategy integrating progressive loading and biological support may better address the multifactorial nature of the condition.

The most relevant clinical observation emerging from this case series is the marked improvement observed across patient-reported outcomes and objective strength measures. The magnitude of change in PRTEE scores, together with the improvement in dynamometric parameters, suggests not only symptom reduction but also meaningful functional recovery. At the individual-patient level, all 10 patients exceeded the 14.8-point MCID threshold for total PRTEE, with reductions ranging from 29 to 85 points. This supports the clinical relevance of the observed short-term within-patient improvement; however, this MCID threshold was derived at a 3-month follow-up in an external LET cohort and should therefore be interpreted cautiously when applied to the present Day-51 assessment (30 days after injection) [10]. Exploratory correlation analyses suggested that residual pain and functional limitation were positively associated at follow-up, whereas pain and function showed inverse associations with mean maximal strength. However, given the very small sample size (n = 10) and the wide 95% confidence intervals, these relationships were imprecisely estimated and should be regarded as hypothesis-generating rather than as evidence of robust, causal, or predictive associations.

The robustness of the main continuous-outcome findings was further examined using exact Wilcoxon signed-rank tests because the very small sample limited the diagnostic value of the Shapiro–Wilk test. The non-parametric sensitivity analyses yielded conclusions concordant with the primary paired *t*-tests: improvements in PRTEE pain, PRTEE function, mean maximal strength, and maximal strength remained statistically significant (all exact *p* = 0.002), whereas maximal strength duration remained non-significant (exact *p* = 0.160). This agreement indicates that the conclusions for these outcomes were not dependent on the normality assumption of the paired *t*-test. It should not, however, be interpreted as overcoming the imprecision, limited generalizability, or threats to causal inference inherent in an uncontrolled case series of 10 patients.

The role of f-ESWT within this protocol deserves particular attention. Rather than acting solely as an analgesic modality, f-ESWT may activate biological processes relevant to tendon healing, including mechano-transduction, neovascularization, and modulation of cellular activity [12,13,14]. This initial phase may therefore contribute to create a more favorable biological context for subsequent rehabilitation and tissue adaptation.

From a biological perspective, collagen peptide-based injection represents a distinct therapeutic concept compared with other orthobiologic treatments such as platelet-rich plasma (PRP). While PRP primarily acts through the release of growth factors and modulation of local signaling pathways, collagen-derived peptides may provide structural substrates and biochemical stimuli that support fibroblast activity and extracellular matrix synthesis [15,16,17,18,19]. This mechanism may be particularly relevant in chronic partial tendon tears, where matrix remodeling becomes a central therapeutic target. Recent pilot studies investigating collagen-based injections in tendon disorders, including rotator cuff pathology and LET, have reported encouraging clinical outcomes, supporting the plausibility of this approach [18,28].

Another clinically relevant aspect of the protocol is the use of US guidance. US allows accurate identification of tendon pathology and precise intralesional delivery of the injective treatment, improving procedural reliability and reducing uncertainty regarding target localization [20,29]. This diagnostic–procedural integration is particularly valuable in partial CET tears, where structural abnormalities and symptom distribution may not fully overlap.

The concordance of findings across patient-reported outcomes, provocative clinical testing, and objective strength assessment documents a consistent pattern of short-term within-patient improvement across multiple outcome domains. However, in the absence of a control or comparator group, this pattern should not be interpreted as evidence of a treatment effect attributable to the multimodal intervention.

## 5. Limitations

Several limitations must nevertheless be acknowledged. The retrospective design, small sample size, and absence of a control group preclude causal inference and limit generalizability. One of the 11 enrolled patients (9.1%) did not attend T1 and was excluded from the complete-case analysis. Although this participant’s age and baseline total PRTEE score were within the ranges observed among the 10 analyzed patients, the absence of follow-up outcome data means that outcome-dependent loss cannot be excluded. If nonattendance was related to lack of improvement or clinical worsening, the complete-case analysis may have overestimated the observed benefits; therefore, the direction and magnitude of potential attrition bias remain uncertain. In particular, the observed changes cannot be distinguished from alternative explanations such as the natural history of the condition, regression to the mean, contextual or placebo effects, or the contribution of concurrent rehabilitation, activity modification, and other components of the multimodal management pathway. The very small sample size also limits the precision of the exploratory correlation estimates, as reflected by their wide confidence intervals, and precludes robust inference regarding associations among outcomes. In addition, the multimodal structure of the protocol does not allow determination of the relative contribution of each intervention component, although this approach more closely reflects real-world clinical rehabilitation practice than isolated single-intervention models. Moreover, T1 was performed on Day 51, 30 days after injection, and should be interpreted as an assessment of short-term clinical responsiveness rather than as evidence of sustained efficacy or tendon healing. The individual PRTEE responder analysis used a 14.8-point MCID derived at 3-month follow-up in an external LET cohort; its application at T1 (Day 51, 30 days after injection) should therefore be regarded as a pragmatic benchmark rather than a time-specific validated cutoff for the present assessment. The relatively short follow-up period also prevents conclusions regarding long-term outcomes, recurrence, or structural tendon evolution over time. Furthermore, the author-developed ordinal scale used to grade provocation-test severity has not undergone formal validation, and intra-observer reliability was not assessed for either the clinical or ultrasound examinations, which is relevant in follow-up studies. Finally, although ultrasound guidance improves procedural accuracy, it remains at least partially operator-dependent.

Although the exact Wilcoxon sensitivity analyses produced conclusions concordant with the paired *t*-tests, they do not overcome the imprecision and limited generalizability arising from the very small sample size.

Despite these limitations, the present study documents the feasibility and tolerability of a structured and integrated management approach for chronic LET with structural tendon damage, together with a consistent pattern of short-term clinical improvement during the observation period. This conceptual framework may help inform future prospective evaluations of stage-adapted multimodal strategies integrating mechanical and biological principles within tendon rehabilitation.

## 6. Conclusions

In this retrospective case series, patients with chronic LET and partial CET tears showed significant short-term improvements in pain, function, and wrist extensor strength following a pathophysiology-driven sequential multimodal strategy combining f-ESWT, progressive therapeutic exercise, and US-guided intralesional injection of LWPs. From a clinical perspective, this multimodal approach appears feasible and well tolerated and is consistent with contemporary principles of interventional physiatry, in which US-guided procedures are integrated with active rehabilitation rather than used as stand-alone interventions.

These exploratory findings show that collagen-based injection can be incorporated within a structured and stage-adapted tendon rehabilitation pathway; however, they do not establish the efficacy of collagen-based therapy, the causal effect of the multimodal intervention, or the relative contribution of its individual components. This framework may warrant further investigation in patients with persistent symptoms and structural tendon damage, in whom conventional conservative strategies may provide incomplete recovery.

Further randomized controlled studies with larger cohorts, longer follow-up, and comparative treatment arms are required to confirm these findings, clarify the specific contribution of each intervention component, and better define the optimal clinical indications for collagen-based interventions in LET.

## Figures and Tables

**Table 1:** Eligibility criteria used for participant selection in the study population.

Inclusion Criteria	Exclusion Criteria
Age > 18 years	Systemic rheumatologic disorders
Lateral elbow pain lasting at least 3 months	Previous elbow surgery
Reproducible pain on at least one standard provocation test (Cozen, Mill, or Maudsley)	Neurological conditions potentially affecting upper limb strength or sensory function
Ultrasound evidence of common extensor tendon (CET) tendinopathy with partial-thickness tear	Previous intratendinous or peritendinous injections in the affected area
Ability to complete the entire treatment protocol, including rehabilitation and clinical reassessment	

Abbreviation: CET, common extensor tendon.

**Table 2:** Author-developed four-level ordinal scale for grading provocation-test severity.

Score	Clinical Interpretation
0	Absence of signs
1+	Mild signs
2+	Moderate signs
3+	Severe signs

**Table 3:** Baseline characteristics of the study population.

Variable	Value
Number of patients enrolled	11
Number of patients analyzed	10
Mean age (years)	48.2
Dominant side affected	90%
Sex	5 males, 5 females

**Table 4:** Individual changes in total PRTEE score and MCID responder status.

Patient	PRTEE Total T0	PRTEE Total T1	Improvement (T0 − T1)	MCID Responder
P1	88	15	73	Yes
P2	84	41	43	Yes
P3	90	5	85	Yes
P4	87	20	67	Yes
P5	89	60	29	Yes
P6	89	9	80	Yes
P7	82	5	77	Yes
P8	74	39	35	Yes
P9	81	7	74	Yes
P10	94	19	75	Yes

Abbreviations: PRTEE, Patient-Rated Tennis Elbow Evaluation; MCID, minimal clinically important difference. Total PRTEE score = pain subscale + function subscale (0–100). Improvement was calculated as T0 − T1. Responder status was defined as an improvement of ≥14.8 points [10].

**Table 5:** Statistical outcomes, effect estimates, distributional checks, sensitivity analyses, and exploratory correlations.

Outcome	T0 (Mean ± SD)	T1 (Mean ± SD)	Mean Change (95% CI)	Cohen’s d_z (95% CI)	Paired *t*-Test *p*
PRTEE pain score	44.3 ± 2.5	13.1 ± 11.2	31.20 (22.22–40.18)	2.48 (1.18–3.76)	<0.0001
PRTEE function score	41.5 ± 3.8	8.9 ± 7.6	32.60 (26.70–38.50)	3.95 (2.05–5.84)	<0.0001
Mean maximal strength (kg)	−4.80 ± 1.60	−0.19 ± 1.48	4.61 (2.89–6.32)	1.92 (0.84–2.98)	0.0002
Maximal strength (kg)	−4.67 ± 2.01	−0.66 ± 1.30	4.02 (2.36–5.68)	1.73 (0.71–2.72)	0.0004
Maximal strength duration (s)	−0.14 ± 0.63	0.14 ± 0.56	0.28 (−0.30 to 0.86)	0.35 (−0.30 to 0.98)	0.300
**Distributional checks and exact Wilcoxon sensitivity analyses**					
**Outcome**	**Median paired change (IQR)**		**Range**	**Shapiro–Wilk W (*p*)**	**Wilcoxon W (exact *p*)**
PRTEE pain score	35.5 (23.75–40.75)		7–43	0.855 (0.067)	0 (0.002)
PRTEE function score	35.0 (24.75–39.25)		20–42	0.856 (0.068)	0 (0.002)
Mean maximal strength (kg)	4.77 (2.96–6.47)		0.85–8.03	0.955 (0.732)	0 (0.002)
Maximal strength (kg)	4.79 (1.98–5.99)		0.28–6.48	0.887 (0.158)	0 (0.002)
Maximal strength duration (s)	0.31 (0.03–0.59)		−1.37–1.50	0.958 (0.763)	13 (0.160)
**Ordinal clinical outcome**					
**Outcome**	**T0 median (IQR)**	**T1 median (IQR)**	**Hodges–Lehmann reduction (95% CI)**	**Wilcoxon W**	***p***
Clinical test score	3.0 (3.0–3.0)	0.5 (0.0–1.0)	2.5 (2.0–3.0)	0	0.002
**Exploratory correlations at T1**					
**Variables**			**Spearman rho**	**95% CI**	***p***
PRTEE pain vs. PRTEE function			0.881	0.467–0.978	0.0008
PRTEE pain vs. mean maximal strength			−0.659	−0.923 to 0.027	0.038
PRTEE function vs. mean maximal strength			−0.606	−0.907 to 0.104	0.064

Abbreviations: PRTEE, Patient-Rated Tennis Elbow Evaluation; SD, standard deviation; CI, confidence interval; IQR, interquartile range. Positive changes indicate clinical improvement. Cohen’s d_z represents the mean paired change standardized by the SD of the within-subject difference scores. Shapiro–Wilk results are reported descriptively because of the limited power of normality testing with n = 10. Continuous-outcome Wilcoxon signed-rank tests were exact and two-sided; W denotes the smaller of the positive- and negative-rank sums. Correlation analyses were exploratory.

## Data Availability

The data supporting the findings of this study are included within the article. Additional anonymized data may be made available from the corresponding author upon reasonable request. Individual patient-level data are not publicly available due to privacy restrictions and compliance with the General Data Protection Regulation (GDPR).

## Abbreviations

The following abbreviations are used in this manuscript:

LET	Lateral Elbow Tendinopathy
LWPs	Low-Molecular-Weight Peptides
PRTEE	Patient-Rated Tennis Elbow Evaluation
f-ESWT	Focused Extracorporeal Schock Wave Therapy
CET	Common Exstensor Tandon
US	Ultrasound
MCID	Minimal Clinically Important Difference
SD	Standard Deviation
PRP	Platelet-Rich Plasma
